# Membrane attack complexes, endothelial cell activation, and direct allorecognition

**DOI:** 10.3389/fimmu.2022.1020889

**Published:** 2022-09-23

**Authors:** Guiyu Song, Shaoxun Wang, Mahsa Nouri Barkestani, Clancy Mullan, Matthew Fan, Bo Jiang, Quan Jiang, Xue Li, Dan Jane-wit

**Affiliations:** ^1^ Section of Cardiovascular Medicine, Dept of Internal Medicine, Yale University School of Medicine, New Haven, CT, United States; ^2^ Department of Obstetrics and Gynecology, Shengjing Hospital of China Medical University, Shenyang, China; ^3^ Department of Surgery, Yale University School of Medicine, New Haven, CT, United States; ^4^ Department of Immunobiology, Yale University School of Medicine, New Haven, CT, United States; ^5^ Department of Vascular Surgery, The First Hospital of China Medical University, Shenyang, China; ^6^ Department of Nephrology, The First Hospital of China Medical University, Shenyang, China; ^7^ Department of Cardiology, West Haven VA Medical Center, West Haven, CT, United States

**Keywords:** complement, endothelial cell, allorecognition, antibody-mediated rejection, transplant

## Abstract

Endothelial cells (ECs) form a critical immune interface regulating both the activation and trafficking of alloreactive T cells. In the setting of solid organ transplantation, donor-derived ECs represent sites where alloreactive T cells encounter major and minor tissue-derived alloantigens. During this initial encounter, ECs may formatively modulate effector responses of these T cells through expression of inflammatory mediators. Direct allorecognition is a process whereby recipient T cells recognize alloantigen in the context of donor EC-derived HLA molecules. Direct alloresponses are strongly modulated by human ECs and are galvanized by EC-derived inflammatory mediators.

Complement are immune proteins that mark damaged or foreign surfaces for immune cell activation. Following labeling by natural IgM during ischemia reperfusion injury (IRI) or IgG during antibody-mediated rejection (ABMR), the complement cascade is terminally activated in the vicinity of donor-derived ECs to locally generate the solid-phase inflammatory mediator, the membrane attack complex (MAC). Via upregulation of leukocyte adhesion molecules, costimulatory molecules, and cytokine trans-presentation, MAC strengthen EC:T cell direct alloresponses and qualitatively shape the alloimmune T cell response. These processes together promote T cell-mediated inflammation during solid organ transplant rejection.

In this review we describe molecular pathways downstream of IgM- and IgG-mediated MAC assembly on ECs in the setting of IRI and ABMR of tissue allografts, respectively. We describe work demonstrating that MAC deposition on ECs generates ‘signaling endosomes’ that sequester and post-translationally enhance the stability of inflammatory signaling molecules to promote EC activation, a process potentiating EC-mediated direct allorecognition. Additionally, with consideration to first-in-human xenotransplantation procedures, we describe clinical therapeutics based on inhibition of the complement pathway. The complement cascade critically mediates EC activation and improved understanding of relevant effector pathways will uncover druggable targets to obviate dysregulated alloimmune T cell infiltration into tissue allografts.

## Introduction

Human ECs comprise a barrier interface regulating activation and recruitment of lymphocytes. In the setting of solid organ transplantation, donor ECs represent sites for initial alloantigen encounter by recipient alloreactive T cells. Donor ECs in humans express both MHC class I and II molecules as well as sufficient costimulatory molecules to enable direct allorecognition. In direct allorecognition, alloimmune T cells recognize antigen in the context of EC-derived HLA molecules. Major alloantigens including class I and II HLA molecules mediate strong type 1 responses, and the strength and quality of these responses are modulated by inflammatory mediators expressed by cognate ECs.

Complement-derived mediators are well known to modulate the direct alloresponse. Complement are evolutionarily ancient immune proteins that allow host recognition of foreign surfaces like donor ECs. In solid organ transplantation, complement proteins become targeted for activation on donor ECs during perioperative ischemia reperfusion injury (IRI) and antibody-mediated rejection (ABMR). When activated on ECs, complement proteins undergo a series of proteolytic cleavages, resulting in the formation of heterodimeric protein complexes with novel enzymatic activities enabling formation of inflammatory mediators. Anaphylatoxins, C3a and C5a, as well as membrane attack complexes (MAC) are inflammatory mediators generated following complement activation. Complement-derived inflammatory mediators promote EC activation and enhance EC-mediated TCR : MHC interactions, costimulatory processes, and T cell recruitment. Improved understanding of the immune mechanisms surrounding complement-mediated EC activation in the setting of direct allorecognition will inform therapies to block EC:lymphocyte interactions and will improve outcomes for transplant recipients.

This review will principally focus on MAC and its attendant molecular mechanisms inducing EC activation to modulate direct allorecognition. We first review complement activation pathways forming MAC, modes of allorecognition, and mechanisms for resisting MAC-induced cell death. We then describe experimental methodologies for studying the biological effects of MAC and MAC-induced signaling pathways. We focus on recent work elucidating MAC-induced ‘signaling endosomes’ that sequester and enhance the stability of pro-inflammatory mediators to promote EC activation. In light of recent first-in-human xenotransplant procedures, we close with a description of therapies focused on complement inhibition and their clinical applications in solid organ transplantation.

## The complement cascade

**Figure 1:**
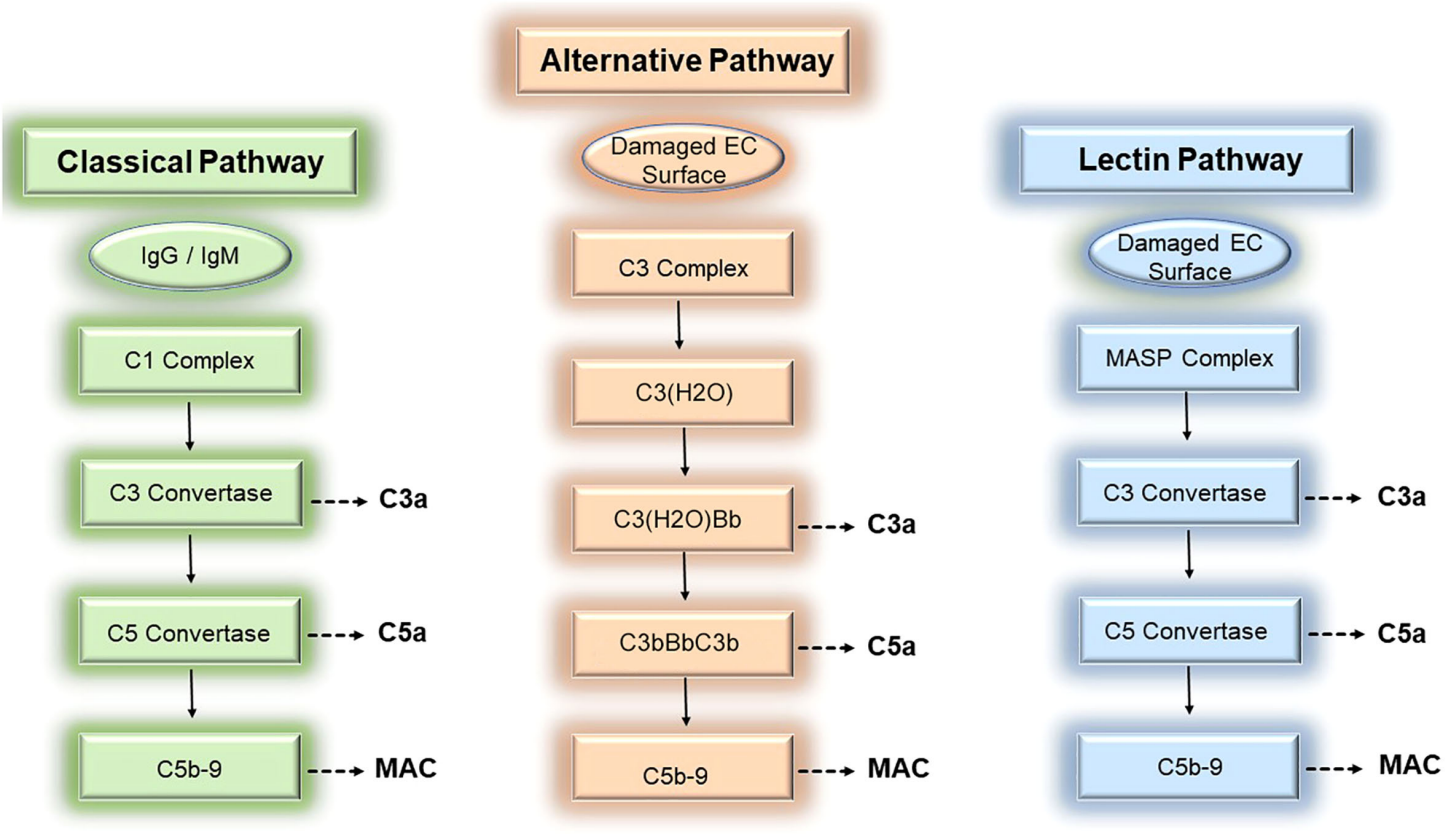
Three pathways of complement activation. The complement cascade may be activated by donor endothelial cells via 3 separable pathways, the classical (left), alternative (middle), and lectin (right) pathways, to mediate rejection of tissue allografts.

The complement system is an evolutionarily conserved system of effectors, split products, and regulatory proteins enabling host recognition of endogenously altered, damaged, or foreign surfaces (1). Following activation by donor ECs, the complement system generates inflammatory mediators, anaphylatoxins, and membrane attack complexes (MAC) that modulate direct allorecognition. We review the 3 established pathways for complement activation below (**Figure 1**).

The complement system is comprised of ~30 soluble- and surface-bound proteins, 9 of which are complement effector proteins. Complement effector proteins, termed C1-C9, are principally produced by the liver and circulate as inactive zymogens. Initially identified as a heat-labile component of normal plasma, complement effectors were renamed C1 to C9 by the World Health Organization in 1968 to reflect the order by which these proteins become activated rather than the order whereby these proteins were discovered (2). Complement effectors, C1-C9, circulate as inactive zymogens and become activated on donor ECs during ischemia reperfusion injury (IRI) and antibody-mediated rejection (ABMR). During these conditions, surface-bound Abs including IgM or IgG bind to post-ischemic neo-antigens (3–5), MHC molecules (6, 7), or non-HLA alloantigens (8, 9) expressed on donor ECs. EC-bound Abs subsequently trigger activation of circulating complement effectors via the classical complement pathway.

The classical pathway involves successive formation of 4 heterodimeric complexes to generate complement-derived inflammatory mediators. The first complex, the C1 complex, contains the pattern recognition receptor, C1q, whose globular head binds to the Fc region of pentameric IgM and monomeric IgG (10, 11) during IRI and ABMR. In addition to Abs, C1q also binds a plethora of EC-derived molecules including various damage-associated molecular patterns (DAMPs, 12, 13), apoptotic cells (14, 15), and altered self-proteins (16–20). Upon ligand binding, C1q becomes complexed with the C1r and C1s proteases to form a C1q-binding tetramer, C1r2C1s2 (21–23). The activated C1 recognition complex subsequently promotes proteolytic cleavage of C4 and C2 whose split products form a second protein complex comprised of C4bC2a heterodimers. C4bC2a heterodimers are a C3 convertase that acquires the ability to proteolytically cleave C3. Cleavage of C3 by the C3 convertase generates the soluble inflammatory mediator, C3a, and a split product, C3b. C3b binds to the C3 convertase to form a third protein complex, C4bC2aC3b, which becomes a C5 convertase with the ability to cleave C5. The C5 convertase mediates cleavage of C5 to generate the potent anaphylatoxin, C5a, as well as the split product, C5b. C5b heterodimerizes with terminal complement proteins C6, C7, to form a ring-like substrate that interacts with the surface lipid bilayer of target cells like donor ECs (24). C5b-7 is a MAC initiator complex that subsequently recruits C8. C8 in turn binds to and accelerates the polymerization of C9 monomers (24). Following binding to C8, C9 monomers recruited from soluble pools in sera undergo unidirectional, clockwise polymerization to form a pore-like structure of 90-110Å in diameter and permeable to molecules of 10-17 kD (25–28). Successful insertion of the C5b-9 complex into donor EC membranes results in formation of MAC, the fourth complement protein complex. MAC are composed of 13-18 C9 molecules (27); trimeric C8 composed of α, β, and γ subunits (29); and the initiator C5b-7 ring which becomes a stoichiometrically minor component of the completed transmembrane structure (27, 28). Following MAC insertion into EC surfaces, MAC cause non-cytolytic EC activation as described in the subsequent section.

Complement activation may be triggered by 2 additional pathways, the lectin pathway and the alternative pathway, both of which have been implicated in vascular rejection of tissue allografts and will be described briefly here. In the lectin pathway, a recognition complex containing mannose-binding lectins (MBLs) or ficolins act as pattern recognition receptors canonically sensing carbohydrate and molecules containing an acetyl determinant like N-acetylglucosamine (GlcNAc) and N-acetylgalactosamine (GalNAc, 30–32). MBLs/ficolins become complexed with mannose-associated serine proteases (MASP1/2/3, 33, 34) that, analogous to C1r/s, sequentially cleave C4 and C2 to generate the second protein complex, C4b2a, that functions as a C3 convertase. Following activation by the MASP recognition complex, the lectin pathway proceeds to generate the same downstream complexes as the classical pathway above including the C5 convertase (C4bC2aC3b) and MAC (C5b-9). Activation of the lectin pathway has been linked to IRI (35, 36), and polymorphisms in the MBL2 pattern recognition receptor, is significantly associated with development of ABMR (37).

The third complement activation pathway, the alternative pathway, has been widely implicated in IRI (38–41). C3 is the most abundant complement protein in plasma, circulating at a concentration of ~1.2mg/mL, and various C3 cleavage products centrally mediate inflammation. C3 molecules undergo tick-over, or constitutive spontaneous hydrolysis, to form biologically active C3(H2O) molecules at a basally low rate of ~1-2 molecules per hour (42). Foreign surfaces like donor ECs dramatically accelerate tick-over, allowing formation of threshold quantities of C3(H2O) that bind to and cleave factor B, forming a C3(H2O)Bb complex that functions as a C3 convertase (43–46). The C3 convertase activity of C3(H2O)Bb cleaves C3 to generate C3a and the split product, C3b. C3b subsequently binds to Bb to form new a protein complex, C3bBbC3b, containing 2 molecules of C3b complexed with Bb. The stability of these complexes is enhanced by properdin/factor D. The C3bBbC3b complex acquires C5 convertase activity and cleaves C5 to form C5a and C5b, the latter of which becomes part of the MAC initiator complex, C5b-7. Similar to the classical or lectin pathways above, C3a, C5a, and MAC are generated as inflammatory mediators through successive proteolytic amplification. Similar to the classical and lectin pathways where gene polymorphisms have been linked to patient outcomes polymorphic variation in C3 has shown associations with ABMR in numerous studies (47–50).

## Complement split products as inflammatory mediators

Complement effectors are highly conserved among metazoans of both invertebrate and vertebrate species. Among the most ancient complement proteins are the early complement effectors, C1-C5, whose evolutionary ages range between 500-1000 million years and whose split products show biological activity. Split products derived from early complement effectors include soluble-phase anaphylatoxins, C3a and C5a, and solid-phase opsonins, iC3b, C3b, and C3d, which covalently bind to donor ECs during IRI and ABMR. A large body of evidence ascribes an inflammatory role for soluble complement mediators in immune cell activation which includes EC:T cell interactions. In addition to opsonizing foreign material, C3a and C5a bind to cognate GPCRs, C3aR and C5aR, respectively, to transmit inflammatory signals to target cells like donor ECs. C3a binds to C3aR, and C5a binds to 2 C5a receptor isoforms, C5aR1 and C5aR2, showing distinct cellular localization and having opposing signaling effects. Knockout mouse models involving C3 and C5 have demonstrated a strong role for anaphylatoxins in mediating myeloid cell recruitment to transplanted tissues as well as roles in recruitment of CD4+ T cells. It is worth noting that there is substantial evidence that C3 and C5, may be also produced by parenchymal and immune cells and that these proteins may become cleaved intracellularly to mediate immune signaling. The roles of intracellularly-derived C3a and C5a and their roles in T cell activation have been comprehensively reviewed elsewhere (51).

Humans express 4 complement receptors, termed CR1-CR4 which variably bind to C3 cleavage products including C3b, iC3b, C3d, C3dg. In particular, iC3b/C3b/C3d form covalent attachments to host proteins, damaged erythrocytes, and Fc receptors of Abs and importantly show binding to CR2. CR2 is heavily expressed on follicular dendritic cells and B cells and mediates pathological B cell activation in various disease settings including viral infection and ABMR (52).

## Autologous endothelial cells resist MAC-induced cell death

As opposed to early complement components, the terminal complement effectors forming MAC, C6-C9, emerged later in evolution (1). Genetic deficiencies of terminal complement components are widely recognized as conferring increased risk for infection by encapsulated bacterial, in particular Neisseriae, and has strongly influenced clinical guidelines regarding vaccination strategies involving multiple pathogens (53). Genetic loss of C9 confers a 700-fold increased risk for Neisserial meningitis (54), suggesting evolutionary development of MAC as a defense mechanism against infection by such pathogens.

While seminally studied for their roles in mediating cytolysis (2), MAC formed from autologous complement proteins from the same species elicit EC activation without substantial EC death *in vivo* (reviewed in 55, 56). In contrast to *in vitro* models showing that MAC may induce cell death of up to ≥50% of treated cells, complement activation in patients, even during severe or terminal instances of ABMR, show disproportionately low frequencies of donor EC cell death in relation to the degree of complement activation. This process also holds true for SARS-CoV-2 infection where ECs show non-cytopathic infection (57) despite strong MAC deposition on target tissues (58, 59); and systemic lupus erythematosus (SLE) where complement-mediated endothelitis occurs without widespread vessel rarefaction (60).

Seminal studies characterizing the physiologic function of MAC employed anucleated red blood cells or xenogeneic substrates like bacteria, as target cells (2). In these studies, the pore-forming capability of MAC which since have been well characterized ultrastructurally and on an atomic scale (24–29) were definitively demonstrated, an effect causing target cell lysis. However, resistance to MAC-induced cell death in nucleated, autologous cells was initially recognized in the 1980s (61–64), and since this time, at least 4 molecular mechanisms enabling target cell survival have been described.

In contrast to anucleated cells and foreign pathogens, autologous cells may constrain MAC-induced cytolysis in a species-specific manner. This phenomenon, known as homologous restriction, is mediated by complement regulatory proteins (65). Many proteins within the complement system contain complement control protein (CCP) domains consisting of conserved repeats that direct interactions among proteins in the complement system. The GPI-anchored proteins, CD55 and CD59, blocking generation of C5b and polymerization of C9, respectively, contain recursive CCP domains that limit assembly and facilitate removal of MAC from the cell surface (66). CCP domains, also known as “Sushi” domains were recently exploited in a bioinformatics approach to identify a new complement regulator, CSMD1, which was found to block MAC assembly at the level of C7 (67) and was implicated in complement activity and infertility *in vivo* and in a patient cohort carrying a non-synonymous CSDM1 mutation (68). MAC regulatory proteins expressed in autologous, nucleated cells thus act as scavengers for limiting the assembly and surface removal of MAC, enabling autonomous cells to resist osmotic lysis. As described below, transgenic knockins of CD46 and CD55, CCP-containing proteins, were used in porcine renal and cardiac xenografts implanted in humans (69, 70).

As a second MAC regulatory mechanism, C5b-9 insertion into target EC membranes is blocked via binding to blood-based chaperones, clusterin (71–73, aka, apolipoprotein J) and vitronectin (74, aka S protein). The C5b-7 ring interacting with target surfaces like donor ECs (24) promotes oligomerization of at least three C9 molecules (75) to form the terminal complement complex, C5b-9. Excess C5b-9 molecules bind to C9-binding chaperones and become trapped in a transition state that is non-permissive for their insertion into target membranes, thereby resulting solubilization (55, 76). Soluble terminal complement complexes, called, Sc5b-9, spatially constrains the radius of MAC assembly to the vicinity of Ab-bound donor ECs. Levels of Sc5b-9 are dramatically elevated during immune responses, and Sc5b-9 has been widely investigated as a biomarker reflecting tissue-bound levels of MAC (77). The biophysical underpinnings for the cell non-autonomous constraint of MAC activity by C9-binding chaperones has been widely investigated, in part due to the notion that the endogenous MAC inactivators, clusterin or vitronectin, could be therapeutically exploited to subvert excess complement activity (reviewed in 78–80).

Exovesiculation or shedding of surface-bound MAC represents a third mechanism by which autologous, nucleated cells survive MAC-induced lysis (81–83). Extracellular vesicles (EVs) are a heterogeneous group of cell-derived vesicles including exosomes and microvesicles. Exosomes and microvesicles are vesicular structures released extracellularly from living cells following MAC deposition on EC surfaces. Exosomes are spherical vesicles of 30-120nm in size that are produced via inward invagination of late endosomal membranes to form multivesicular bodies (83). Multivesicular bodies subsequently fuse with the plasma membrane to release their intraluminal contents including exosomes extracellularly. In contrast, microvesicles are ~100-1000nm in diameter, are derived from cell membranes, and contain high levels of surface-derived EC molecules including integrins, CD40L, and complement regulatory proteins CD55 and CD59 (83, 84). EVs including exosomes and microvesicles can be released by ECs (85, 86) and PMNs (87) and efficiently activate complement *in vitro* (85, 87) and thus it is postulated that EV membranes may serve as decoy substrates to limit MAC deposition on host tissues.

Finally, elimination of MAC from donor EC surfaces may reduce MAC to sub-lytic levels, thereby enabling intracellular coping pathways to restore homeostasis. As reviewed elsewhere (88), various intracellular pathways promote resistance to MAC-induced cytolysis, a process that has been explored in the setting of tumor cell evasion from complement-dependent cytotoxicity. Intracellular pathways conferring resistance to MAC-induced cytolysis include pathways related to intracellular trafficking of MAC (81, 82), calcium handling (89), MAPKs (82, 90), heat shock proteins (91), and NF-κB (92, 93). Together, the 4 regulatory mechanisms above contribute to survival of autologous, nucleated cells from MAC-induced lysis.

## Experimental models of MAC assembly

Evolutionary development of mechanisms for resisting MAC-induced cytolysis implies separable, non-cytolytic effects of MAC on target cells and provides a basis for studying inflammatory signaling in MAC-respondent cells like donor ECs. To study effects of non-cytolytic MAC, at least 5 experimental models for inducing MAC assembly have been developed. A widely used system for assembling MAC on nucleated cells involves the use of xenogeneic antisera, e.g., rabbit sera overlay on mouse target cells. This widely-used protocol has allowed discovery of various MAC-induced inflammatory pathways operative in immune cells including neutrophils, dendritic cells, and ECs (88) including NLRP3 inflammasome activation (94, 95). A second model for eliciting MAC assembly on nucleated cells incorporates stepwise addition of terminal complement proteins at defined stoichiometric ratios to allow formation of MAC in cell culture (96). This protocol, theoretically recapitulating homologous restriction, has been adapted for use *in vivo* (97). A third protocol for experimental MAC assembly involves the use of zymosan-activated serum. Zymosan is primarily composed of the highly cross-linked polysaccharides alpha-D-mannan, beta-D-glucan, and other minor polysaccharide polymers derived from yeast (S. cerevisiae). Yeast-derived zymosan potently activates all 3 complement pathways and has been widely used to study downstream effects of anaphylatoxins on immune cells such as neutrophils and macrophages (28).

Recently, human sera have been exploited as a source for complement proteins to enable formation of autologous MAC on human ECs. In a model of ABMR, human ‘high’ panel reactive antibody (PRA) sera taken from allo-sensitized transplant candidates were used to elicit autologous MAC deposition on human ECs (98). PRA sera induced non-cytolytic MAC on human ECs in an IgG-dependent manner *in vitro* and *in vivo*, facilitating identification of MAC signaling pathways including non-canonical NF-κB (98–100), NLRP3 inflammasome (101, 102), and canonical NF-κB (101). These pathways collectively contributed to EC activation and enhanced EC-mediated direct allorecognition. In a model of IRI, human sera used as a source for complement proteins deposited non-cytolytic MAC on human ECs in an IgM-dependent manner following *in vitro* anoxia of human ECs or coronary artery segments (102). Using this model, EC-mediated direct alloresponses promoted selective expansion of T peripheral helper (T_PH_) cells but not T follicular helper (T_FH_) cells in an IL-18-dependent manner to promote B cell activation and *de novo* DSA within donor tissues. Various model systems have been developed to assemble non-cytolytic MAC *in vitro* and *in vivo* and have shown utility in defining MAC signaling effects across numerous clinical settings.

## Human ECs mediate direct allorecognition

**Figure 2:**
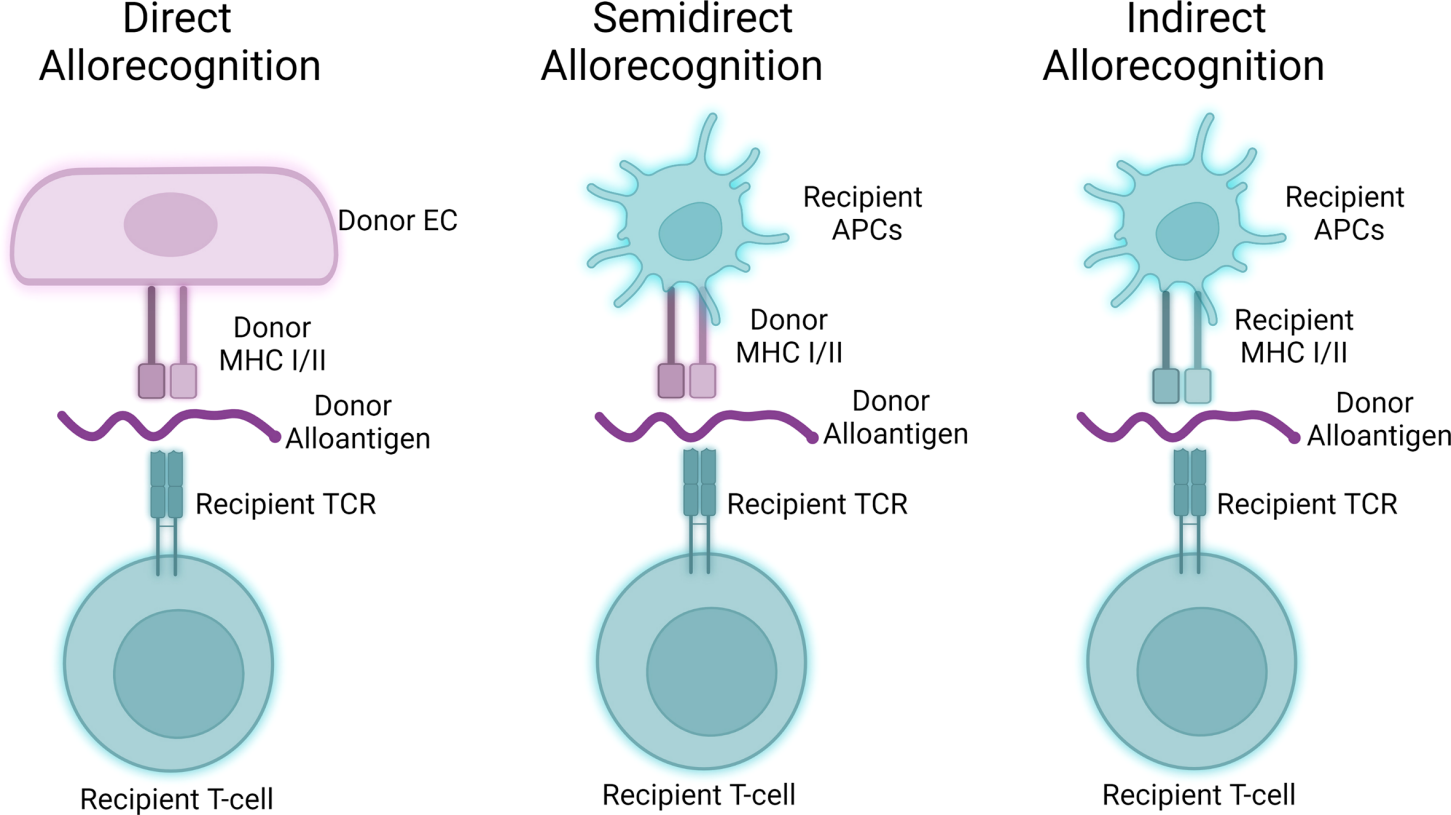
Three pathways of allorecognition. Donor-derived alloantigens may activate allogeneic T cells via three separable pathways. Human endothelial cells may act as antigen presenting cells to participate in direct allorecognition responses (left), while murine hosts primarily participate in semidirect and indirect allorecognition (middle, right) through interactions involving professional antigen presenting cells.

Alloantigen encounter may occur via 3 pathways including direct allorecognition, indirect allorecognition, and semi-direct allorecognition (**Figure 2**; 103). In direct allorecognition, the antigen presenting cells (APCs) are the donor-derived cells that present donor MHC: peptide complexes to the host T cells. In indirect allorecognition, the donor-derived antigens are acquired and processed by the recipient APCs. The recipient APCs then present the donor antigens to host T cells. Semidirect allorecognition occurs when MHC: peptide complexes shed from donor-derived cells become captured by recipient APCs (2). The recipient APCs then present MHC: peptide complexes to host T cells. T cell activation is restricted by the donor MHC molecules in direct allorecognition, whereas the T cell activation is restricted by recipient MHC molecules in indirect allorecognition. Because the intact donor-derived MHC molecules are presented by the recipient APCs in semidirect allorecognition, T cell activation is restricted by donor MHC molecules in this context (104).

Human ECs are functionally different from mouse ECs in allograft rejection as they are capable to provide costimulatory signaling to activate T cells via direct allorecognition. This is because of the unique expression pattern in antigen-presenting molecules and co-stimulators in human endothelial cells. Human ECs not only express both MHC I and MHC II but also constitutively express co-stimulators inducing LFA-3, ICOS ligand, programmed cell death protein Ligand-1 (PDL1), 4-1BB ligand, and OX40-ligand. Murine ECs unlike human ECs lack the ability to strongly elicit direct alloresponses due to the lack of expression of key costimulatory molecules, most saliently CD58/LFA3 (105). These co-stimulators in human ECs allow direct recognition and activation of central and effector memory T cells (106–108). The primary vascular cell type responsible for direct allorecognition in graft arteries is ECs as vascular smooth muscle cell expresses rather a low level of HLA molecules (105). Following direct allorecognition, humoral- and surface-derived signals cause EC-mediated allostimulation. While MHC class I/II strongly induce type 1, i.e., IFN-γ, responses from T cells, EC:T cell crosstalk results in EC elaboration of various cytokine factors including TGF-β and IL-6 that may further influence differentiation of T cells activated through direct allorecognition. Allostimulation following direct allorecognition is further enhanced by processes affecting ECs including local complement activity as described in the following section. In ABMR, the major cell infiltrate in graft artery intima are T cells, most of which demonstrate a type 1 effector profile characterized by interferon-gamma (IFN-γ) production. There is a significant amount of IFN-γ production in the recovered T cells isolated from lesions in allograft vasculopathy, and IFN-γ is necessary and sufficient to drive cardiac allograft vasculopathy, a condition characterized by pathologic vascular changes in transplant patients with chronic ABMR (109).

Major or minor alloantigen mismatch in a heterotopic heart transplantation model in mice elicits T cell-mediated rejection principally via indirect allorecognition. To test EC-mediated direct allorecognition, a response operative in human tissues, our group has developed humanized mouse models. In a human artery xenograft model, human coronary artery segments are subjected to various treatments simulating IRI (99, 110, 111) or ABMR (98–102, 112) prior to surgical implantation into descending aortae of immunodeficient SCID/beige mice engrafted with human T and B cells. Human artery xenografts subjected to IRI potentiate the T cell-meditated allograft injury in the human artery xenograft model. Because the vascular smooth muscle cannot activate allogeneic T cells and the absence of leukocytes in the donor grafts activated human ECs appears to be the dominant APCs in this system (99, 110, 111). Indeed, human artery xenografts subjected to IRI or ABMR treatments show non-cytolytic MAC assembly on intimal ECs, a process causing EC activation and resulting in potentiated alloimmune T cell activation. As myeloid cells including antigen presenting cells like dendritic cells do not engraft in SCID/beige mice, the potentiated T cell activation in this model is primarily driven by direct allorecognition and formation of *de novo* donor specific antibody in this model occurs within allograft tissues and not the spleen (110). The grafted artery shows intimal expansion characterized by an infiltration of CD45RO+ T cells, the formation of a human EC-lined microvessel, and the presence of VMSCs. These observations highly mimic the clinical features of graft arteriosclerosis in allograft vasculopathy (109). To further uncover the underlying mechanisms, we found that formation of MAC (99) and MAC-induced inflammatory mediators in human ECs including ZFYVE21 (100), NLRP3 (101, 102), IL-15 (112), and IL-18 (110) potentiates frequencies of CD4+ T cells producing IFN-γ.

In a second humanized model, human umbilical vein endothelial cells (HUVECs) are suspended on collagen-fibronectin gel matrices and implanted subcutaneously in SCID/beige mice. Three to four weeks post-implantation, HUVECs self-organize into perfused microvascular networks anastomosing with murine vasculature (113). By transducing HUVECs with various overexpression, dominant negative constructs (101), or CRISPR/Cas9 gene edits (114) prior to implantation, this model allows interrogation into how immune molecules including MAC-induced signaling proteins in ECs affect direct allorecognition. Murine and humanized models enable the study of indirect and direct allorecognition responses by ECs, respectively.

## MAC-induced EC activation and direct allorecognition

Donor ECs are a principal cell type affected by MAC in ABMR and IRI. via activation on donor ECs, MAC centrally governs EC:T cell interactions, and MAC formation on ECs is prognostic for, diagnostic for, and a therapeutic target for lymphocyte-mediated injury of tissue allografts in these settings. The use of antisera or human sera to assemble MAC on nucleated cells as described above has enabled elucidation of MAC signaling pathways and their effects on direct allorecognition.

MHC-derived alloantigens strongly mediate type 1 responses, i.e., IFN-γ production, and the strength and quality of this response was found to be modulated by MAC. Non-cytolytic MAC deposition on human ECs upregulated adhesion molecules including VCAM-1 and E-selectin, and inflammatory gene transcripts encoding chemokines like CCL5 and CCL20 and cytokines like IL-6 (98). In EC:T cell cocultures, MAC-treated ECs increased frequencies of adherent CD4+CD45RO+ memory T cells (T_mem_) under conditions of postcapillary venular shear stress and non-specifically enhanced bulk cytokine effector responses involving IFN-γ, IL-4, and IL-17 in EC:T cell cocultures. *In vivo*, human coronary artery segments exposed to PRA and implanted in immunodeficient mice engrafted with human T and B cells showed increased infiltration of intimal IFN-γ+ T cells causing neointimal expansion, i.e., cardiac allograft vasculopathy (98, 99). Thus, MAC broadly enhances the recruitment and strength of effector activity of T_mem_ following EC-mediated direct allorecognition.

In two separable processes, MAC may qualitatively shape allostimulation, the immune response occurring following direct allorecognition. First, MAC may induce differential expansion of T cell subsets following direct allorecognition. A survey of costimulatory molecules in MAC-treated ECs showed that MAC upregulated PD-L2 and ICOS-L, cognate receptors for PD-1 and ICOS, respectively (110). PD-1 and ICOS are heavily expressed on 2 recently described subsets, T peripheral helper (T_PH_) and T follicular helper (T_FH_) cells. These subsets express PD-1 and ICOS and elaborate IL-21 to promote B cell maturation and Ab responses. MAC-treated ECs were found to selectively enhance expansion of T_PH_ cells in an IL-18-dependent manner, a process eliciting production of *de novo* DSA within inflamed peripheral vessels. Mechanistically, MAC induced NLRP3 inflammasome activity in ECs, resulting in IL-18 release. IL-18 binds to its cognate receptor, IL-18R, which was selectively expressed on T_PH_ cells co-expressing CCR2 but not TFH cells co-expressing CXCR5. This differential expression of IL-18R caused selective expansion of T_PH_ but not T_FH_ cells. CCR2+ T_PH_ cells infiltrated into IRI tissues to mediate B cell maturation and formation of *de novo* DSA in human artery xenografts *in vivo*, and mass cytometry (CyTOF) analysis of T_mem_ in renal transplant recipients with IRI showed selective expansion of T_PH_ but not T_FH_ cells in peripheral circulation (110). This study demonstrated that MAC could qualitatively shape the T cell response by promoting differential expansion of a defined T cell subset following direct allorecognition.

Secondly, MAC-induced IL-1β resulting from inflammasome activity enhances allostimulation by expanding cytotoxic T lymphocytes. In an autocrine fashion, MAC-induced IL-1β was found to increase production of IL-15 which became associated with the IL-15 receptor α chain (IL-15Rα) at the EC surface (112). This complex trans-presented IL-15 to effector memory CD8^+^ T cells to induce their proliferation and to allow these cells to become cytotoxic T lymphocytes (CTLs), characterized by production of granzyme B and perforin. Trans-presentation of IL-15 by ECs to T cells additionally increased trans-endothelial migration by activating CD11a/CD18 and motility. While there is a propensity for activation and recruitment of CD8^+^ T cells relative to CD4^+^ T cells by EC trans-presentation of IL-15, both populations respond to some degree to surface IL-15/IL-15Rα complexes (112). With IL-15 blockade by monoclonal antibody, co-cultured CD4^+^ and CD8^+^ T cells lose polyfunctionality, an effect which is only partially reproduced by IL-1 receptor antagonism. A likely explanation for this is the evolution of other cytokines by the co-cultured T cells themselves that signal through canonical NF-κB, such as tissue necrosis factor α, and are sufficient to produce surface IL-15/IL-15Rα (115). This study shows that MAC assembly on ECs could induce sequential production of IL-1β and IL-15 to qualitatively modulate the direct alloresponse. Accumulated data show that MAC may modulate both the strength and quality of direct alloresponses of alloimmune T cells.

## MAC-induced signaling endosomes

**Figure 3:**
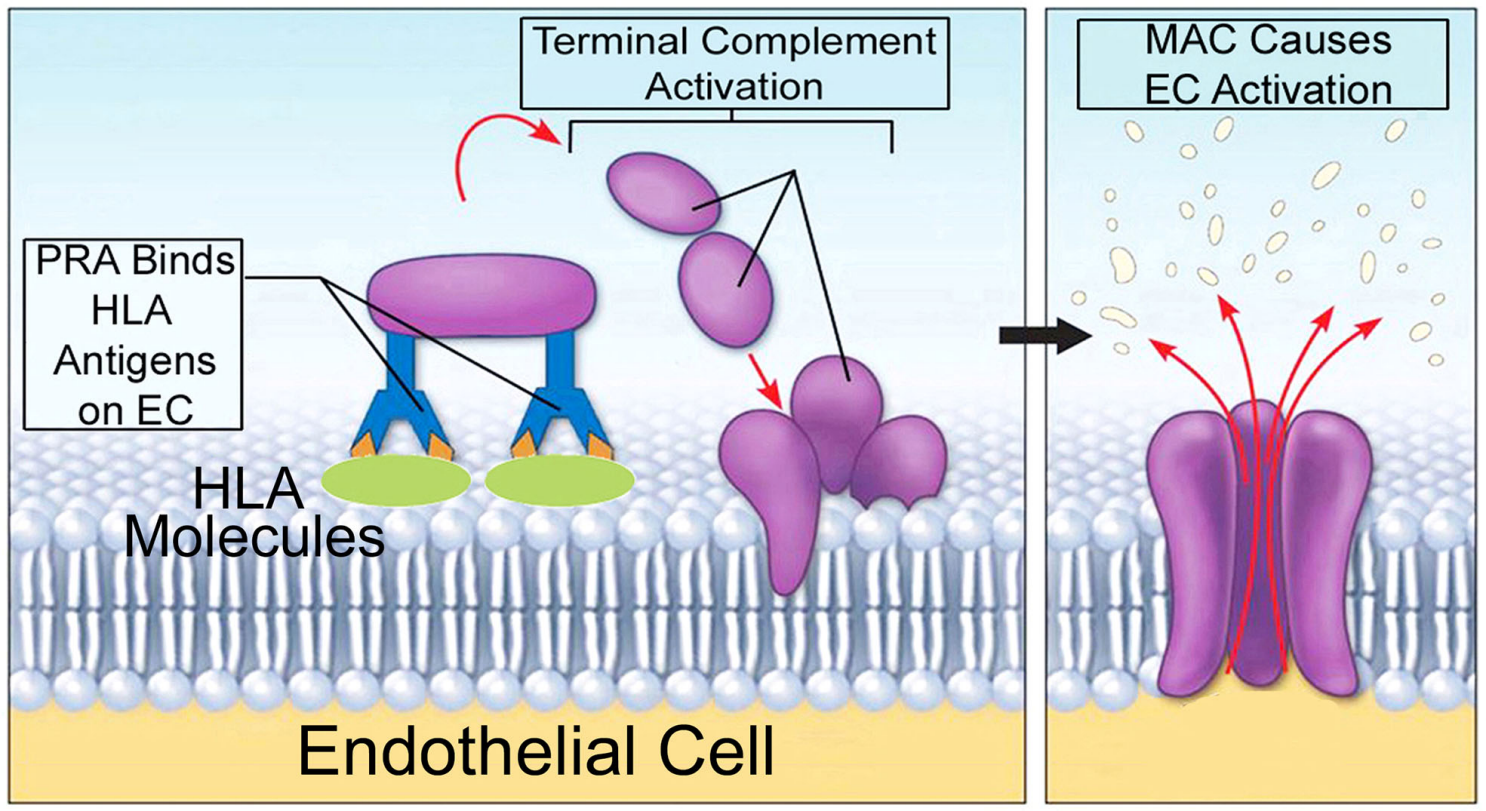
A model for autologous alloantibody-induced MAC assembly. 'High' panel reactive antibody (PRA) sera is obtained from transplant candidates and contain high titers of alloantibodies binding to many HLA specificities. PRA mediates binding of donor specific alloantibody to surface MHC I/II molecules on human ECs. This activates complement components within PRA sera, causing terminal activation of the classical complement pathway and formation of non-cytolytic MAC on target ECs. Human-derived MAC on human ECs causes EC activation without inducing cell lysis.

Companion studies have elucidated inflammatory signaling pathways induced by MAC that modulated the direct alloresponses above. Using human PRA sera to assemble autologous MAC on human ECs (**Figure 3**), it was found that MAC and not IgG or anaphylatoxins, activated non-canonical NF-κB, a pathway marked by NF-κB-inducing kinase (NIK). Activation of non-canonical NF-κB by MAC occurred within 15-30 min (98, 100) in a TRAF3-independent manner (99), features starkly contrasting with described pathways of non-canonical NF-κB involving ligand:receptor interactions occurring in 18-24 hr and requiring TRAF3 degradation. The unusual pattern of non-canonical NF-κB activity induced by MAC, coupled with the observation that MAC became internalized to form a MAC+Rab5+ intracellular compartment (100), led to the hypothesis of the ‘signaling endosome’ enabling MAC-induced inflammatory signaling.

Drug, siRNA, and dominant negative treatments blocking MAC+Rab5+ endosome formation reduced inflammatory gene transcripts and attenuated EC-mediated generation of IFN-γ+ T_mem_ in EC:T cell cocultures (100). This remarkable finding indicated that MAC did not elicit NF-κB activity or EC activation from the cell surface. Rather, MAC rapidly underwent clathrin-mediated endocytosis and transfer to Rab5+ vesicles, forming MAC+Rab5+ endosomes. This compartment contained ‘signaling endosomes’ which were found to sequester and post-translationally enhance the stability of signal-activating molecules by protecting these same molecules from proteasome degradation (100).

**Figure 4:**
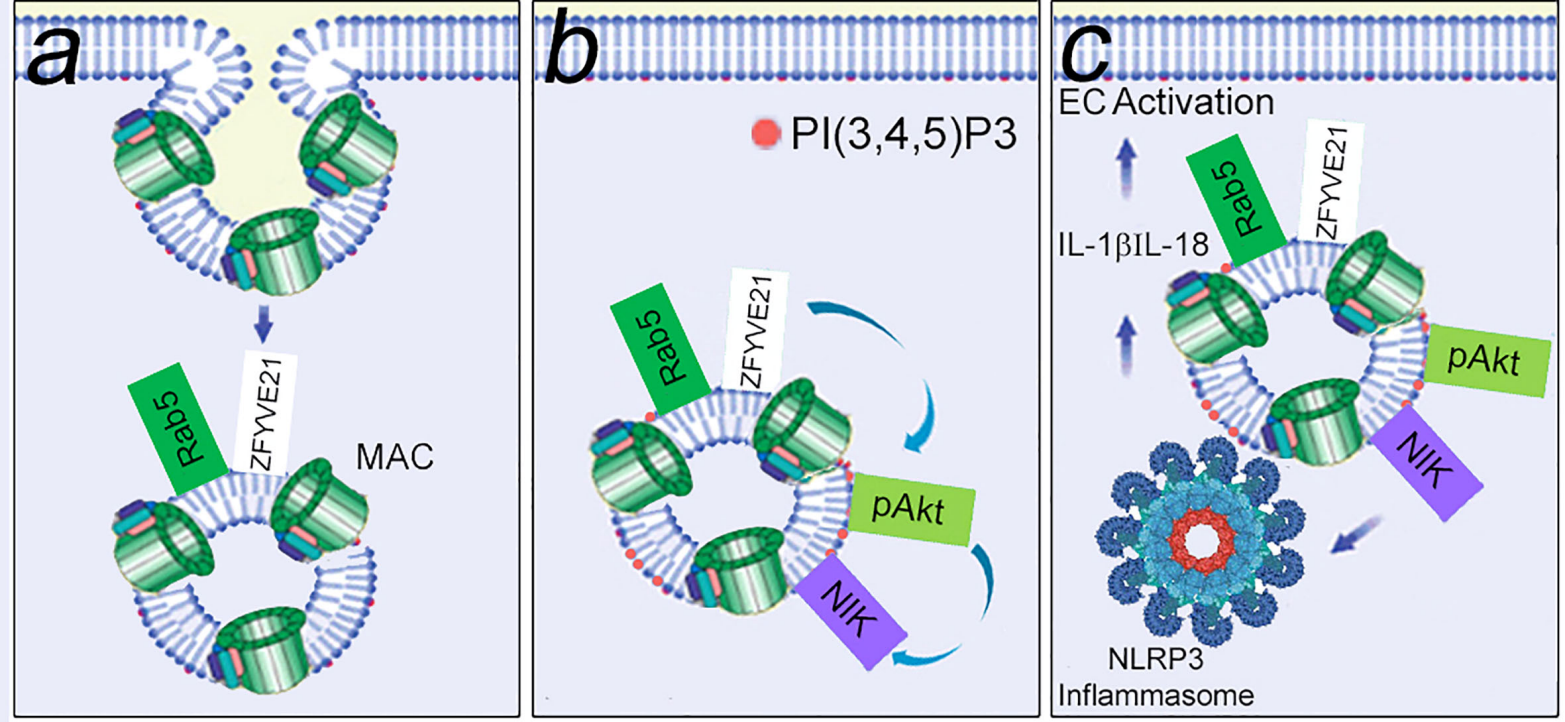
MAC-induced signaling endosomes cause EC activation. Non-cytolytic MAC assembled on EC surfaces rapidly undergoes clathrin-mediated endocytosis to form MAC+Rab5+ signaling endosomes that sequester signal-activating elements. In a Rab5-dependent manner, signaling endosomes post-translationally stabilize ZFYVE21, a Rab5 effector. **(A)** ZFYVE21 recruits SMURF2, an E3 ubiquitin ligase, to mediate degradative removal of PTEN from signaling endosomes, causing enrichment for PI(3,4,5)P3 and recruitment of phosphorylated Akt (pAkt, **B**). This process sequentially activates non-canonical NF-κB [marked by NIK (NF-kB Inducing Kinase)], NLRP3 inflammasomes, and IL-1β- mediated canonical NF-kB **(C)**.

As an extension of the signaling endosome hypothesis, we reasoned that these same structures were enriched in signal-activating proteins. We devised a protocol for proteomic analysis of FACS-sorted MAC+Rab5+ endosomes, and this protocol facilitated unbiased identification of ZFYVE21 which we found was a novel Rab5 effector (100). ZFYVE21 is a conserved molecule localized to early endosomes initially implicated in tumor cell migration (116) but whose functions remain poorly understood. Our group found that ZFYVE21 regulated lipid and protein remodeling of MAC+Rab5+ endosomes to cause sequential pAkt recruitment and enhanced NIK stability. Post-translationally stabilized levels of NIK promoted recruitment of NLRP3 from the endoplasmic reticulum and caspase-1 from the cytosol to appose these molecules on signaling endosomes. As a result, inflammasome activity characterized by cleaved caspase-1, occurred, resulting IL-1β-dependent activation of canonical NF-κB. In sum, MAC sequentially activated 3 inflammatory pathways in an inter-dependent fashion. MAC caused early-phase activation of non-canonical NF-κB (**Figure 4**). Non-canonical NF-κB caused assembly of NLRP3 inflammasomes on signaling endosomes within 30 min (101, 102). Endosome-associated caspase-1 activity generated IL-1β, causing late-phase activation of canonical NF-κB at the ≥4 hr timepoint (101). Inhibition of MAC via a monoclonal anti-C5 Ab (110), pharmacologic blockade of ZFYVE21 induction (100), or drug-induced attenuation of NLRP3 oligomerization (112) reduced EC-mediated direct allorecognition and EC injury in a humanized mouse model. These studies collectively advanced the hypothesis that non-cytolytic MAC forms signaling endosomes that sequester and enhance the stability of inflammatory signaling proteins.

## Complement-based therapeutics

Recent studies have shown remarkable clinical utility of complement-based therapeutics. Drug development efforts have focused on attenuating salient steps in the complement pathway including protease activation of the C1 recognition complex, early pathway amplification at the level of C3, and terminal pathway activation at the level of C5. We highlight FDA-approved drug inhibitors acting at each of the steps in the complement pathway above. Sutimlimab is a humanized monoclonal antibody targeting C1s, the protease component of the classical complement pathway mediating cleavage of C2 and C4. Sutimlimab recently received FDA approval in Feb 2022 for the indication of cold agglutinin disease, a form of autoimmunehemolytic anemia involving MAC-induced hemolysis (117). Compstatins are cyclic peptides originally derived from a peptide isolated via phage display library (118) and iteratively modified to selectively bind C3 and to inhibit its cleavage by C3 convertases (119). A derivative of this original peptide, pegcetacoplan, received fast-track and orphan drug approval in July 2022 for the indication of paroxysmal nocturnal hemoglobinuria (PNH), a rare genetic disorder caused by aberrant glycosylphosphatidylinositol (GPI) conformation causing deficient expression of CD55 and CD59 and unconstrained MAC-induced hemolysis. Eculizumab is a humanized murine monoclonal antibody against C5, which prevents C5 cleavage and the formation of MAC by any of the three complement pathways. Like pegcetacoplan, eculizumab was granted orphan drug approval for PNH along with atypical hemolytic uremic syndrome, a condition also characterized by MAC-induced hemolytic anemia. Since its approval, eculizumab has gained an additional indication for blocking B cell activation in myasthenia gravis and off-label usage for blocking direct alloresponses in ABMR (120). Recently, a second and presumably more cost-effective long-acting monoclonal C5 Ab, ravulizumab, has also received FDA approval for the indications above. The risk:benefit profiles for the drugs above appear reasonable, and the complement pathway has been shown to be a viable drug target for multiple clinical conditions.

Highlighting its fundamental importance in solid organ transplantation, the complement pathway was identified as a key genetic target in first-in-human porcine xenotransplants. We briefly describe this exciting and rapidly evolving clinical application. Galactose-α-1,3-galactose (α-gal) is a terminal carbohydrate modification present in porcine but not human tissues, and human subjects frequently show high titers of circulating xeno-Abs against this epitope. Xeno-Abs against α-gal cause vascular rejection via hyperacute ABMR, a process mediated by MAC, and this catastrophic condition has limited the utility of xenografts in patients. Formation of α-gal is mediated by the porcine enzyme, α-1,3-galactosyltransferase. In December 2020, the FDA granted a first-of-its-kind approval for intentional genomic alterations (IGA) targeting porcine 1,3-galactosyltransferase. In porcine xenografts, CRISPR-based methods knocking out porcine 1,3-galactosyltransferase was combined with subcapsular autologous thymic tissue implants in a proof-of-principle study to prevent hyperacute ABMR. Two porcine-to-human xenotransplant procedures were performed in two brain-dead patients with end-stage renal failure, and following surgical implantation, over a period of ~2 days the implanted xenografts carrying the α-gal IGA demonstrated improvements in renal function including decreasing creatinine, increasing estimated glomerular filtration rate, and increasing urine production. These improved parameters occurred in the absence of hyperacute ABMR (69). This initial study demonstrated proper functioning of porcine tissue within a human environment in the absence of complement-mediated ABMR.

In a follow-up study involving an orthotopic heart xenograft (70), further IGAs were made to the porcine xenograft to promote xenograft acceptance. CRISPR-mediated gene edits were made to knock out 3 highly immunogenic xenoantigens (galactose-a-1,3-galactose, Sda blood group antigen, and N-glycolylneuraminic acid) and growth hormone receptor to prevent intrinsic xenograft growth. To block complement-mediated injury, knockins were performed for the complement regulatory proteins, CD46 and CD55, as well as EC-derived proteins facilitating direct allorecognition, CD47 and heme oxygenase-1. Complement proteases intersect and show activity against proteins related to coagulation (121), and coagulation factors including thrombomodulin and protein C were knocked in to prevent hypercoagulability linked to inefficient thromboregulation via porcine proteins. Excitingly, the porcine xenograft containing the gene edits above survived 60 days following organ implantation and showed various signs of proper functioning including normal sinus rhythm, cardiac output, longitudinal strain, and left ventricular ejection fraction. The patient’s post-operative course was complicated by oligoanuric renal failure requiring renal replacement therapy, bacterial and fungal peritonitis, and increased titers of the porcine CMV/suid herpesvirus 2, indicating a possible zoonotic infection. Endomyocardial biopsies, though showing signs of injury following the patient’s demise at day post-op day 60, did not show evidence of acute ABMR. These preliminary findings appeared to validate the efficacy of the immunomodulatory IGAs made to the porcine xenograft which included complement regulatory proteins. Genetic supplementation for endogenous complement inhibition through IGA is a newly emerging therapeutic, and the clinical application for this technology in patients appears very promising at the moment.

## Conclusions

In solid organ transplantation, autologous MAC are non-cytolytic and modulate adaptive immunity by enhancing the strength and quality of direct allorecognition by ECs. Highlighting its clinical relevance, MAC formation on ECs is prognostic for, diagnostic for, and a therapeutic target for vascular rejection. Blockade of MAC via pharmaceutical and genetic approaches are emerging as new treatment modalities. Future work elucidating the immune roles of MAC may uncover gene targets enabling blockade of its formation on donor tissues and/or attenuation of its downstream inflammatory pathways.

## Author contributions

MB, SW, CM, MF, BJ, QJ, GS, XL, and DJ drafted the text of this review. MB and DJ produced figures for the manuscript. All authors contributed to the article and approved the submitted version.

## Funding

DJ was supported by grants from the NIH (R01HL141137-01, R01HL141137-04S2), the Veterans Hospital Administration (I01 BX005117), and the American Lung Association (ETRA 736563). JP was supported by grants from the NIH (R01HL-51014).

## Conflict of interest

Work supporting development of humanized mouse models was supported in part through a research grant from Alexion Pharmaceuticals (JP). Work supporting identification of ZFYVE21 was supported in part through a research grant from Abbvie Pharmaceutical Research and Development (JP, DJ).

The remaining authors declare that the research was conducted in the absence of any commercial or financial relationships that could be construed as a potential conflict of interest.

## Publisher’s note

All claims expressed in this article are solely those of the authors and do not necessarily represent those of their affiliated organizations, or those of the publisher, the editors and the reviewers. Any product that may be evaluated in this article, or claim that may be made by its manufacturer, is not guaranteed or endorsed by the publisher.
